# *ZbAGL11*, a class D MADS-box transcription factor of *Zanthoxylum bungeanum*, is involved in sporophytic apomixis

**DOI:** 10.1038/s41438-020-00459-x

**Published:** 2021-02-01

**Authors:** Xitong Fei, Qianqian Shi, Yichen Qi, Shujie Wang, Yu Lei, Haichao Hu, Yulin Liu, Tuxi Yang, Anzhi Wei

**Affiliations:** 1 College of Forestry, Northwest Agriculture and Forestry University, Yangling, Shaanxi China; 2 Research Centre for Engineering and Technology of Zanthoxylum State Forestry Administration, Yangling, Xianyang 712100 China

**Keywords:** Seed development, Reproductive biology

## Abstract

Apomixis is a reproductive model that bypasses sexual reproduction, so it does not require the combination of paternal and maternal gametes but instead results in the production of offspring directly from maternal tissues. This reproductive mode results in the same genetic material in the mother and the offspring and has significant applications in agricultural breeding. Molecular and cytological methods were used to identify the reproductive type of *Zanthoxylum bungeanum* (ZB). Fluorescence detection of the amplified products of 12 pairs of polymorphic SSR primers showed consistent fluorescence signals for mother and offspring, indicating that no trait separation occurred during reproduction. In addition, the cytological observation results showed differentiation of ZB embryos (2n) from nucellar cells (2n) to form indefinite embryonic primordia and then form adventitious embryos (2n), indicating that the apomictic type of ZB is sporophytic apomixis. The MADS-box transcription factor *ZbAGL11* was highly expressed during the critical period of nucellar embryo development in ZB. Unpollinated *ZbAGL11*-OE *Arabidopsis* produced fertile offspring and exhibited an apomictic phenotype. The overexpression of *ZbAGL11* increased the callus induction rate of ZB tissue. In addition, the results of the yeast two-hybrid experiment showed that ZbAGL11 could interact with the ZbCYP450 and ZbCAD11 proteins. Our results demonstrate that *ZbAGL11* can cause developmental disorders of *Arabidopsis* flower organs and result in apomixis-like phenotypes.

## Introduction

The development of plant flower organs is controlled by regulatory genes that control floral organ development by activating or inhibiting the expression of other genes^1–4^. This is the most basic principle of the ABC model of floral organ development in plants, which was originally developed by *George W. Haughn* and *Chris Somerville* to describe the reproductive characteristics of *Arabidopsis thaliana*^5^. The ABC model was modified and improved to become the more comprehensive ABCDE model, which includes five types of MADS-box transcription factors with different functions:^6^ Class A genes control the development of the first and second whorls of petals. The loss of this type of gene causes sepals to develop into carpels and petals to develop into stamens, with mutations including *APETALA1* (*AP1*) and *APETALA2* (*AP2*)^7^. Class B genes control the development of the second whorl of petals and the third whorl of stamens. The loss of B gene function causes petals to develop into bracts and stamens to develop into carpels with mutations such as *APETALA3* (*AP3*) and *PISTILLATA* (*PI*). Class C genes control the development of the third whorl of stamens and the fourth whorl of carpels. Loss of class C gene function causes stamens to develop into petals and carpels to develop into sepals, with mutations including *AGAMOUS* (*AG)*, *SPATULA* (*STP*), and *CRABS CLAW* (*CRC*). Class D genes control the development of the fifth whorl of ovules, and the loss of D gene function can cause the ovule to become a leaf or carpel structure^8^, with mutations such as *AGAMOUS-LIKE 11* (*AGL11*), *SHATTERPROOF1 (SHP1)*, and *SHATTERPROOF2 (SHP2)*. E_1_, E_2_, E_3_, and E_4_ genes (*AGL2*, E_1_; *AGL4*, E_2_; *AGL9*, E_3_, and *AGL3*, E_4_) are involved in the formation of the first calyx whorl, and E_3_ is additionally involved in the formation of the second, third, fourth, and fifth whorls of flower organs^9^. Class D genes exhibit high expression levels in the central tissues of seeds and young fruits and play key roles in ovule development and seed formation^10,11^. Overexpression of the D-type MADS-box transcription factor *AGL11* affects the differentiation of fleshy tissue and the internal structure of tomato fruit^1,10^. Silencing the *SlyAGL11* gene in tomato produces a seedless fruit, demonstrating that *AGL11* plays an important and direct role in seed development in fleshy fruits^1^.

Apomixis is a reproductive mode that does not require the parental gametophyte to produce a zygote but instead results in the direct production of offspring from the mother^12,13^. There are two types of apomixis that differ in terms of the embryo sources and developmental processes: sporophytic apomixis and gametophytic apomixis^14,15^. In sporophytic apomixis, embryo sacs form through meiosis and mitosis in the nucellus, and adventitious embryos are formed from integument cells (somatic cells). Depending on embryo sac origin, gametophyte apomixis can be classified as diplospory or apospory. In diplospory, the embryo sac forms from the megaspore mother cell (2n) through mitosis, and the diploid egg cell (2n) develops directly into an embryo. In apospory, the embryo sac forms from the nucellar cell (2n) through mitosis, and the embryo is formed by the aposporous initial cell (2n) in the embryo sac^16^. Apomixis can fix heterosis for the maintenance of excellent maternal economic traits, making determination of the detailed mechanisms important for theoretical research and breeding applications^16^.

*Zanthoxylum bungeanum* (ZB) is known as Chinese prickly ash and is an important economic tree species of the Rutaceae family. More than 250 species of Chinese prickly ash are widely distributed around the world^17^. The peel of this species is used as a famous traditional condiment and is also a traditional Chinese medicine^18^. ZB is a dioecious plant with both male and female plants, but in nature, male plants are extremely rare. Only female plants are planted in artificial plantations, and there is evidence that the reproductive mode of ZB is apomixis^19,20^. However, the type and molecular mechanism of apomixis in ZB are unclear, limiting its wider application. The MADS-Box transcription factor family is widely involved in the regulation of plant growth and development, especially the development of floral organs. By measuring the gene expression levels and performing cytological analysis, we found that *ZbAGL11*, a member of the MADS-Box transcription factor family, is highly expressed at the critical stage of apomixis in *ZB*. To further investigate the relationship between *ZbAGL11* and apomixis, we assessed the apomictic type of *ZB* and determined the critical period of development. We also tested the function of *ZbAGL11*, and the results of this work provide a reference for further study of the function of *ZbAGL11* and reveal the mechanism of apomixis in this species.

## Results

### Cytological analysis of embryo sac development in ZB

**Fig. 1:**
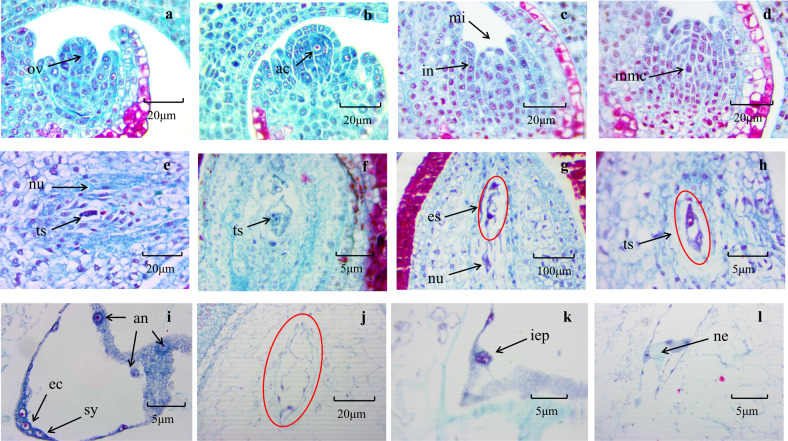
Development of apomictic embryo sacs in *Zanthoxylum bungeanum*. **a** Ovule. **b** Archesporial cell. **c** Integument developing into a micropyle. **d** Megaspore mother cells. **e** Two-nucleus embryo sac. **f** Two-nucleus embryo sac. **g** Disintegration of embryo sac. **h** Nucleus cells form an unreduced embryo sac. **i** The mature embryo sac gradually disintegrates. **j** Degeneration of embryo sac. **k** Early stage of differentiation of nucellus cells. **l** The nucellus cells differentiate to form a nucellar embryo. ac archesporial cell, an antipodal cell, ec egg cell, es embryo sac, hs heart-shaped embryo, iep indefinite embryonic primordia, in integument, mi micropyle, mmc megaspore mother cells, ne nucellar embryo, nu nucellus, ov ovule, sy synergid, ts two-nucleus embryo sac

We followed the development of the ZB embryo sac by sectioning and observing samples embedded in paraffin. The ovule formed a peripheral cell by continuous differentiation (Fig. 1a). When the peripheral cells reached 3–4 layers, archesporial cells began to appear (Fig. 1b). The archesporial cell then underwent meiosis and mitosis steps to form functional megaspores, and the other three megaspores were degraded. The integument also developed, and the two approached one another to form the micropyle (Fig. 1c). Functional megaspores underwent a mitosis step to form a two-nucleus embryo sac (Fig. 1d–f). Subsequently, the embryo sac disintegrated, and a nucellus cell adjacent to the embryo sac then differentiated to form a two-nucleus embryo sac (Fig. 1g, h). After two mitoses, a mature embryo sac of seven cells and eight nuclei was formed (Fig. 1i). The polar nucleus spontaneously developed into endosperm without being fertilized, and the egg cells, synergid cells, and antipodal cells in the embryo sac gradually disintegrated (Fig. 1j). The indefinite embryonic primordia at the bottom of the embryo sac began to differentiate (Fig. 1k), developed into a heart-shaped embryo, and eventually formed an embryo (Fig. 1l). The endosperm began to develop before the embryo sac matured and was not dependent on fertilization.

**Fig. 2:**
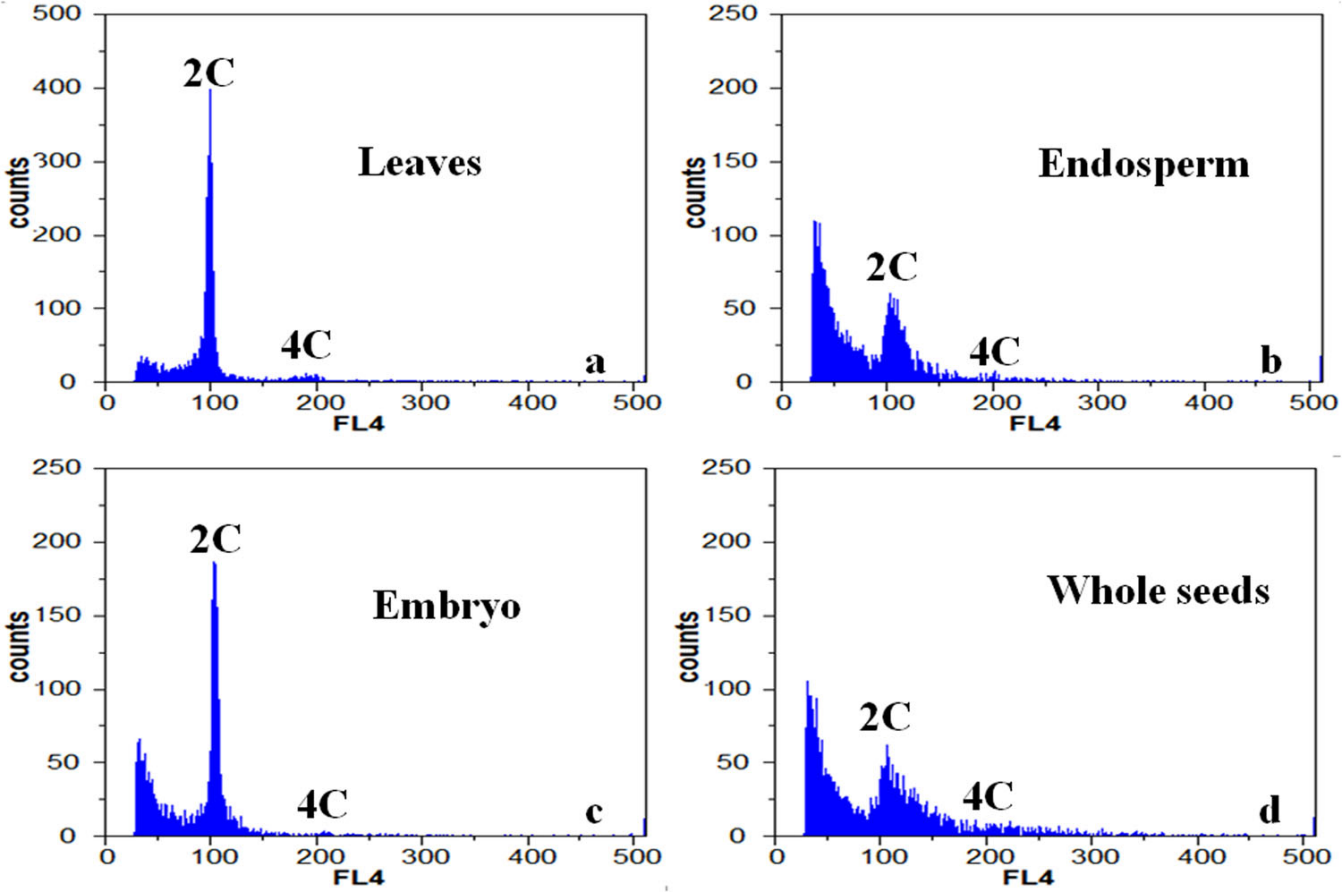
Distribution of DNA contents in different tissues of *Zanthoxylum bungeanum*. **a** Leaves. **b** Endosperm. **c** Embryo. **d** Whole seeds

To further determine the source of the embryo, we next analyzed the relative ploidy of different tissues in the ZB seed^21^. The relative ploidy was determined for the leaf, endosperm, embryo, and whole seed of ZB using flow cytometry (Fig. 2a). Two peaks were detected in the leaves, at 98.72 and 192.91. The ratio of these peaks was 1:1.95, with relative ploidy close to 2 C:4 C. In the leaves, the ratio of the first peak cells was 64.44%, and the ratio of the second peak cells was 4.92%. The observed difference in ploidy was due to the mitosis of the cells, which caused a portion of cells to stay in the G2 phase.

The ploidy of endosperm and somatic cells was consistent with 2n (Fig. 2b, c), and we did not detect 3n endosperm produced by double fertilization (Fig. 2d). The tested samples were obtained from plants cultivated at plantations with only female plants, and all the flowers were bagged to prevent fertilization. Overall, the results show that ZB endosperm (2n) can spontaneously form independent of fertilization, and the ploidy of the endosperm was consistent with that expected for somatic cells (leaf 2n) and was not the 3n endosperm that would be produced by double fertilization.

### Capillary fluorescence electrophoresis

To identify whether trait separation occurred during the propagation of ZB, the amplified products of 48 DNA samples (3 mothers and 45 offspring) were detected by capillary electrophoresis on a 3730Xl gene sequencer. The 5′-ends of each of the 12 upstream SSR (simple sequence repeat) primers were labeled with FAM, so polymorphisms of the amplified product could be detected based on the fluorescent signals. Fluorescence detection of the amplified products of 12 pairs of polymorphic SSR primers showed consistent fluorescence signals for mother and offspring (Fig. 1 and S1, 2), indicating that no trait separation occurred during reproduction and providing additional support for the occurrence of apomixis in ZB.

### Tissue-specific expression and subcellular localization of *ZbAGL11*

**Fig. 3:**
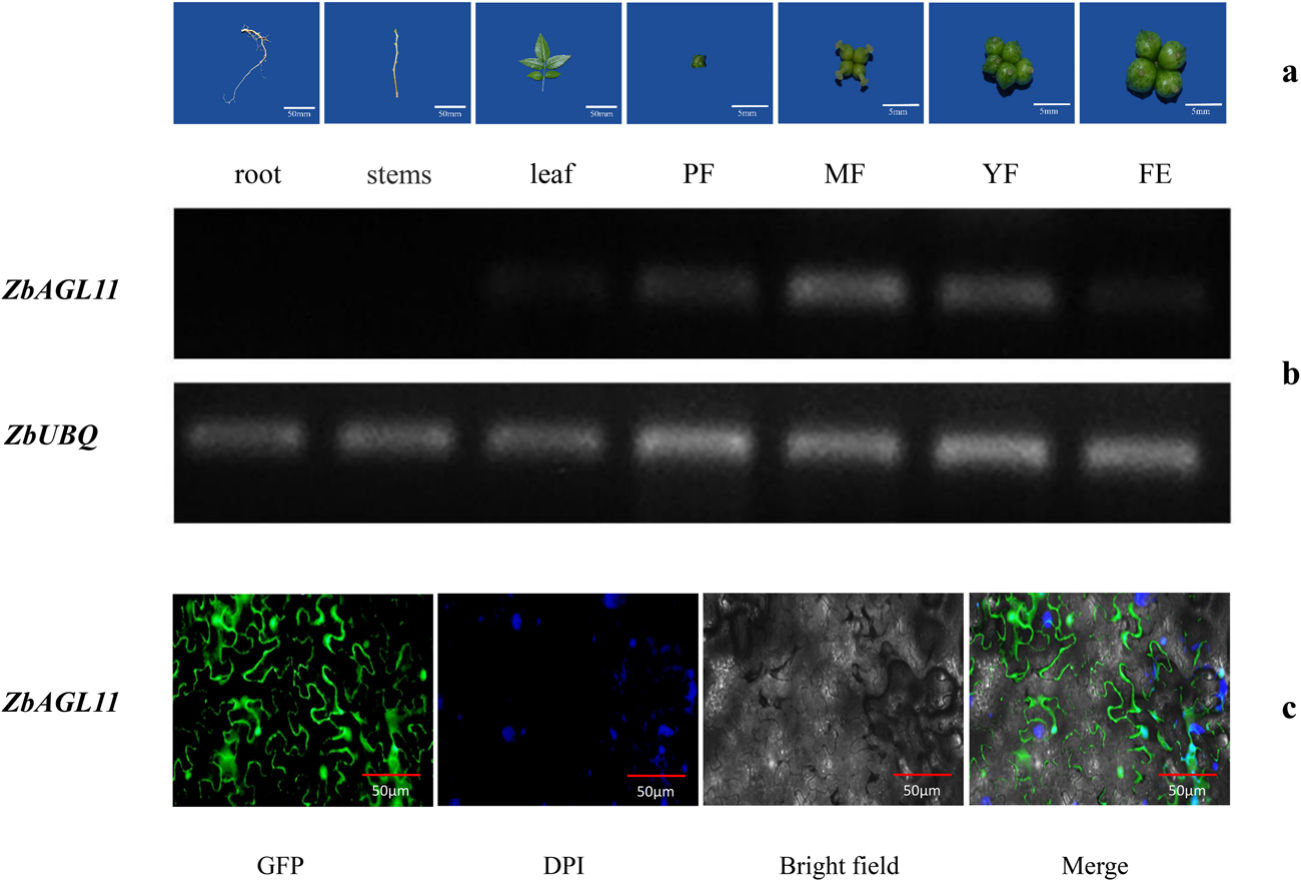
Tissue-specific expression and subcellular localization of *ZbAGL11*. **a** Different tissues of *Zanthoxylum bungeanum* and different stages of fruit development. PF preflowering, MF midflowering, 3 days after flowering, YF young fruit, 7 days after flowering, FE fruit expansion, 15 days after flowering. **b** Tissue-specific expression of *ZbAGL11*. **c** Subcellular localization of *ZbAGL11*

The spatiotemporal expression of *ZbAGL11* was investigated by semiquantitative analysis of different tissues, including roots, stems, leaves, flowers, and fruits (Fig. 3a). The results showed little expression of *ZbAGL11* in roots and stems, a relatively low expression level in leaves, and a relatively high expression in flowers and fruits (Fig. 3b). The relative expression level of *ZbAGL11* was highest in the young fruit stage (YF), corresponding to the apomictic nucellar embryo stage of ZB. To further determine the localization of *ZbAGL11* in the cell, a *ZbAGL11*-GFP fusion vector was constructed. Agrobacterium containing the *ZbAGL11*-GFP fusion vector was injected into leaves of *Nicotiana benthamiana* and cultured for 48 h before observation of the fluorescence signal. Green fluorescence was observed on the cell membrane and nucleus, indicating that these locations may be the site of *ZbAGL11* protein function (Fig. 3c).

### Phylogenetic relationship between *ZbAGL11* and homologous genes

MEGA software was used to analyze the evolution of the AGL11 protein sequences of 37 species, including ZB. According to the analysis, the protein sequence of ZbAGL11 exhibited the highest homology with the genes from *Citrus sinensis* and *Citrus clementina*, which are also members of the Rutaceae family (Fig. S2a). However, there was a significant genetic distance between ZbAGL11 and other species, raising the possibility that the *ZbAGL11* gene may play different functions from the genes in other species. Rosaceae, Leguminosae, and Cruciferae plants were clustered together, indicating that AGL11 was relatively conserved among these families. *ZbAGL11* belongs to class D of the MADS-box transcription factor family, and we also analyzed the evolution of other members of the MADS-box transcription factor family with *ZbAGL11*. As shown in the evolutionary tree (Fig. S2b), *ZbAGL11* (class D) had the highest homology with *AG* (class C). In addition, *SPT* (class C) clustered with *PI* (class B) and *SHP1* (class D), and class A also clustered with classes C, D, and E. This indicates conservation among members of the MADS-box transcription factor family and implies the potential for functional overlap between members.

### *ZbAGL11* overexpression induces dramatic modifications in flower and fruit organization

**Fig. 4:**
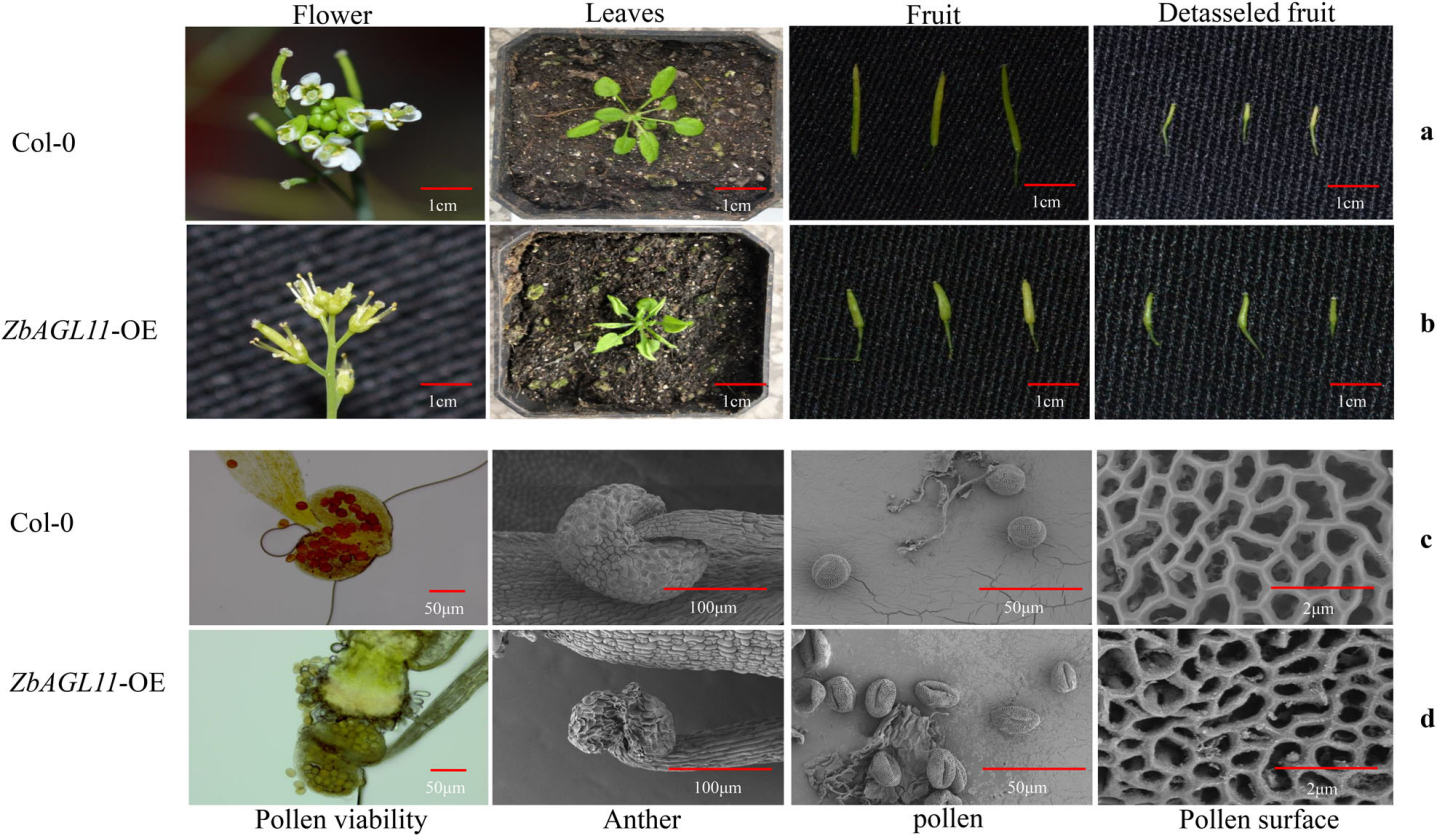
Phenotype and physiology in transgenic Arabidopsis. **a** Flowers, plants and fruit pods of Col-0. **b** Flowers, plants and fruit pods of *ZbAGL11*-OE. **c** Stamen microstructure of Col-0. **d** Stamen microstructure of *ZbAGL11*-OE

To investigate the role of *ZbAGL11* in flower development, we compared the morphologies of sepals, petals, stamens, pistils, and pollens of Col-0 and *ZbAGL11*-OE *Arabidopsis* and evaluated the detasseled performance of plants and pollen viability (Fig. 4). The results showed that overexpression of *ZbAGL11* resulted in atrophy of the sepals (first whorl) and petals (second whorl) of *Arabidopsis thaliana* while significantly increasing the length of stamens (third whorl) and pistils (fourth whorl). Scanning electron microscopy and pollen viability results showed that the overexpression of *ZbAGL11* resulted in sexual reproductive disorders in *Arabidopsis*, including wrinkled anthers, empty pollen, and decreased pollen viability. During the flowering period, the pistils of *ZbAGL11*-OE *Arabidopsis* were significantly longer than those of the wild type, and the pod length was significantly shorter in *ZbAGL11*-OE *Arabidopsis* than in wild-type plants during the fruit ripening period. In addition, after emasculation, the fruit pods of Col-0 *Arabidopsis* withered and fell off at the early stage of fruit development, while the pods of *ZbAGL11*-OE *Arabidopsis* remained able to develop normally after emasculation, but with significantly shortened fruit pods that were thicker in diameter than Col-0.

**Fig. 5:**
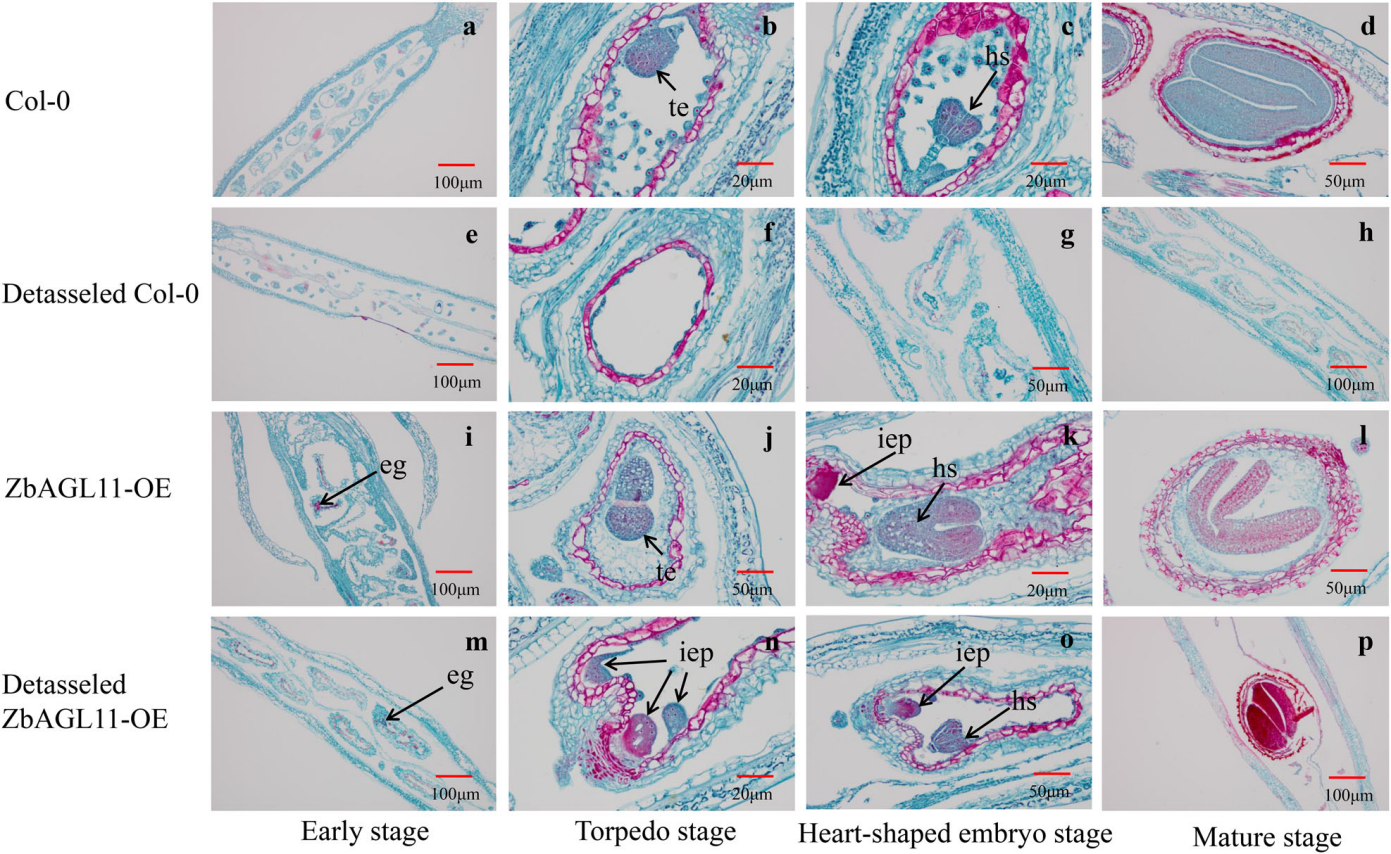
Wild-type (Col-0) and *ZbAGL11*-OE Arabidopsis embryonic development. eg egg, hs heart-shaped embryo, iep indefinite embryonic primordia, te torpedo embryo.

In addition, imaging of paraffin sections was performed to observe the embryonic development of Col-0 and *ZbAGL11*-OE *Arabidopsis* under normal and detasseled conditions. The results showed that *Arabidopsis* Col-0 formed torpedo-shaped and heart-shaped embryos under normal culture conditions that eventually developed into mature embryos (Fig. 5a–d). The embryo and endosperm of Col-0 did not develop normally after detasseling (Fig. 5f–h), but *ZbAGL11*-OE was able to form multiple indefinite embryonic primordia during the torpedo stage after detasseling (Fig. 5n). However, the endosperm could not develop autonomously without the participation of male gametes in *ZbAGL11*-OE. The indefinite embryonic primordia developed into heart-shaped embryos (Fig. 5o) and finally formed seeds. The seed-setting rate of 100 detasseled *ZbAGL11*-OE pods was calculated as only 5% (seed-setting rate = number of mature seeds/number of embryo sacs * 100%).

To confirm the origin of detasseled *ZbAGL11*-OE embryos (2n/n), we next performed flow cytometry experiments on somatic cells of *ZbAGL11*-OE and progenies using Col-0 as a control group. Due to endoreduplication in *Arabidopsis* somatic cells, 2 C, 4 C, 8 C, and 16 C nuclei were present in somatic cells (Fig. S3). The flow cytometry results showed consistent nucleic acid ploidy (diploidy) of the progeny of *ZbAGL11*-OE and *ZbAGL11*-OE with that of Col-0. The results showed that after blocking of sexual reproduction (detasseling), the embryos produced by *ZbAGL11*-OE developed from somatic cells (2n) rather than from egg cells (n). The cytological observations and cell ploidy results indicated that the overexpression of the *ZbAGL11* gene can lead to apomictic traits in *Arabidopsis*, whereas *ZbAGL11*-OE can produce fertile offspring independently of fertilization.

### Expression patterns of MADS-box transcription factors

**Fig. 6:**
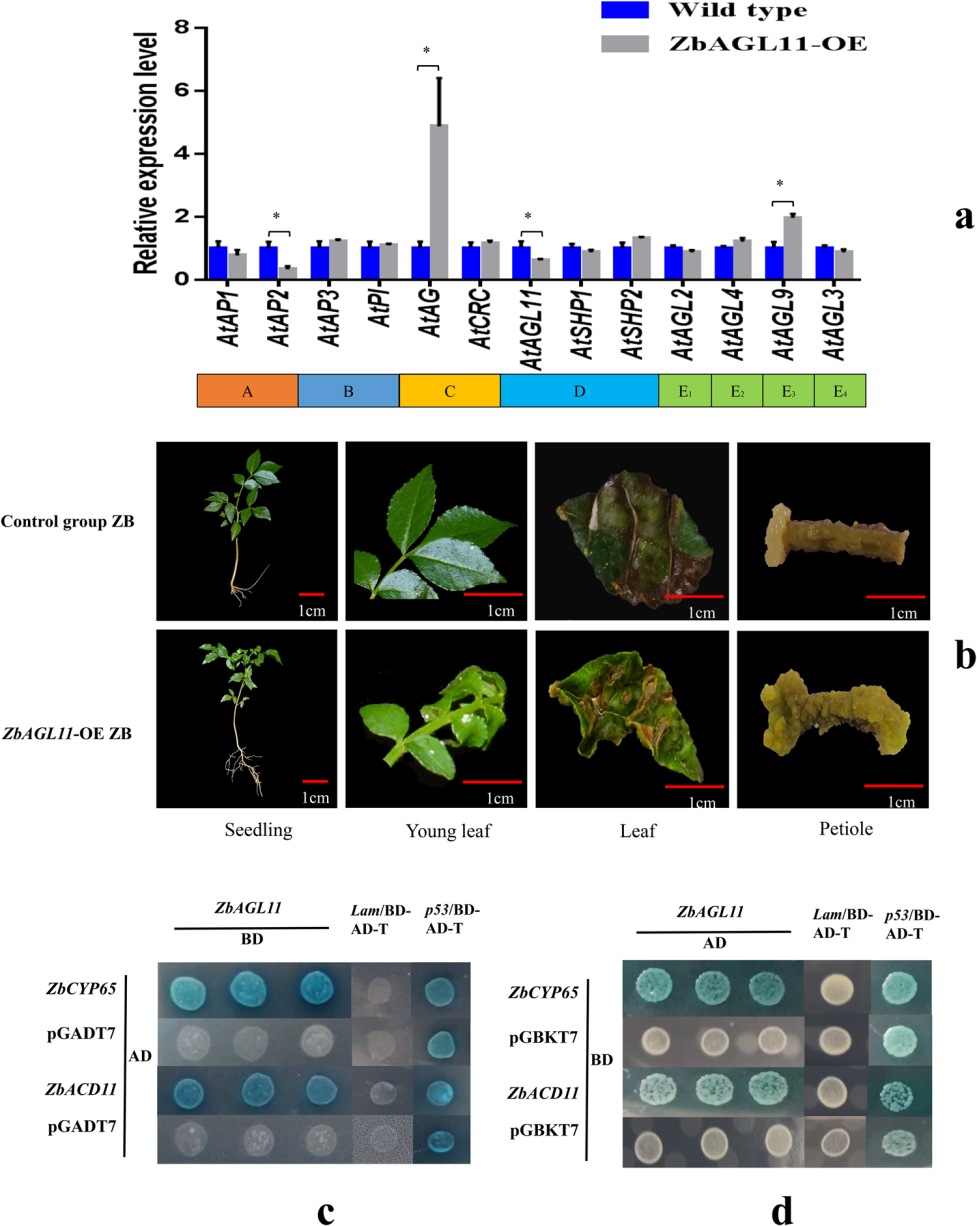
Functional analysis of MADS-box transcription factors. **a** Relative expression levels of MADS-box transcription factors. **b** Phenotype of 35S: *ZbAGL11 Zanthoxylum bungeanum*. **c** Yeast two-hybrid assays for the interaction between *ZbAGL11* and *ZbCYP65* or *ZbACD11*. **d** Yeast two-hybrid rotary validation

To study the effect of *ZbAGL11* on the expression level of Arabidopsis MADS-box transcription factors, we tested the expression levels of 13 genes (including genes from each of the five classes, A, B, C, D, and E) in wild-type cells and cells overexpressing *ZbAGL11*. The results showed that the levels of nine genes were not significantly different between the two materials (Fig. 6a). However, the relative expression levels of *AtAG* (Class C) and *AtAGL9* (Class E_3_) in *ZbAGL11*-OE *Arabidopsis* were significantly higher than those in the wild type, and the relative expression levels of *AtAP2* and *AtAGL11* were significantly lower than those in the wild type.

### Overexpression of *ZbAGL11* phenotype in ZB

The *ZbAGL11* gene was transformed into ZB calli by *Agrobacterium* infection to obtain resistant shoots. The resistant buds were then screened on MS medium containing kanamycin (25 mg/l), and finally, *ZbAGL11*-overexpressing plants were obtained. In the control group, the ZB leaves were flat, and the plants had fewer roots, while *ZbAGL11*-OE ZB leaves were curly, and the plants had more roots (Fig. 6a). Interestingly, leaf curling also appeared in *ZbAGL11*-overexpressing *Arabidopsis*. One plant tissue culture pathway is through somatic embryos, which is similar to the pathway of apomixis. Therefore, using 35 S: ZbAGL11 ZB and control group ZB as experimental materials, studying the differentiation of calli in vitro will help reveal the role of ZbAGL11 in apomixis. Leaf and petiole samples were prepared as plant tissue culture materials using MS medium without added hormones. The control group leaves could hardly produce calli, while 35 S: *ZbAGL11* was able to produce fresh calli, with a callus induction rate of 72.5%. The callus induction rate of the control group petioles was only 15.0%, while 35 S: *ZbAGL11* could induce a large number of fresh callus tissues, with an induction rate of 85.0%. In summary, 35 S: *ZbAGL11* exhibited a significantly higher callus induction rate than the control group, indicating that the *ZbAGL11* gene has a positive role in promoting cell differentiation.

### Yeast two-hybrid assays

To investigate potential protein interactions, a bait vector (pGBKT7-*AGL11*) and a library vector (pGADT7-cDNA) were cotransformed into Y187 yeast competent cells and cultured in SD/-Leu/-Trp-deficient medium. White colonies were transferred to SD/-Leu/-Trp/-Ade/-His/X-α-gal/AbA-deficient medium, blue strains were expanded, and then the plasmids were extracted. The plasmids were then used as templates for pGADT7 vector universal primers for PCR amplification and sequencing. The sequencing results identified the genes on the pGADT7 vector as peptidyl-prolyl cis-trans isomerase CYP65 (*ZbCYP65)* and accelerated cell death 11 (*ZbACD11*). To further verify an interaction between *ZbAGL11* and *ZbCYP65* proteins, we switched the constructs to encode *ZbCYP65* in the pGBKT7 vector and *ZbAGL11* in the pGADT7 vector (Fig. 6c, d). The two new plasmids were then cotransformed into yeast, and the yeast strain was still able to grow and turn blue on SD/-Leu/-Trp/-Ade/-His/X-α-gal/AbA-deficient media, confirming the interaction.

## Discussion

Apomixis does not rely on double fertilization but is the direct differentiation of somatic cells or embryo sacs without meiosis to form embryos. This allows the production of offspring that are identical to the maternal genetic material, bringing new opportunities for agricultural breeding. Most crops are propagated by sexual reproduction, and there are challenges in using conventional breeding methods to transplant apomictic traits into crops. Therefore, there is a need for genetic engineering as a way to transform sexual reproductive potential into crops with apomictic characteristics^22^. It is important to better understand the process and regulatory mechanism of apomixis, as different types of apomixis in different plants may have different regulatory mechanisms. In this study, cytological observation, ploidy identification, and SSR molecular markers were applied, and the results showed that apomixis is the reproductive mode of *ZB*, with sporophytic apomixis identified as the apomictic type. *ZB* and citrus are members of the Rutaceae family, and citrus also utilizes sporophytic apomixis^22,23^, indicating that the type of apomixis may be the same among closely related species, providing a reference for the identification of apomictic types of closely related species.

The ABCDE model is widely accepted as a model of flower development regulation and includes five types of genes classified by function^6^. These genes have functional redundancy in flower organ development and have antagonistic effects^24^. *AGL11*, a class D gene, is indispensable in the development of carpels^25^. The classic ABCDE model describes the function of class D genes as related to the development of ovules, and in Arabidopsis, the ectopic expression of the *AtAGL11* gene can induce the transformation of sepals into carpeloid organ-bearing ovules^26^. In the ABCDE model, *AGL11* genes are grouped into class D based on gene function, and AG genes are grouped into class C. However, *AGL11* (class D) genes have high homology with AG (class C) genes. The class C AG genes may regulate stamens, carpels, and ovules, while the class D AGL11 genes may regulate only the development of ovules. The *ZbAGL11*-OE phenotype has many similarities with that of *Arabidopsis* 35 S: *AG*, including stamen elongation and petal degeneration^27^. However, there were some differences in the phenotypes, with elongated pistils and shortened sepals in *ZbAGL11*-OE and deformed sepals in 35 S: *AG*. In addition, *AGL11*/*SEEDSTICK* can control Arabidopsis fruit size by regulating cytokinin levels and *FRUITFULL*^28^. By examining the expression level of the MADS transcription factor genes, we found that expression of *AtAG* (Class C) and *AtAGL9* (Class E_3_) increased significantly in *ZbAGL11*-OE Arabidopsis, while that of *AtAP2* (Class A) and *AtAGL11* (Class D) decreased significantly. We speculate that *ZbAGL11* can increase the expression levels of *AtAG* and *AtAGL9*, thereby promoting the elongation of stamens and pistils. Based on the close interactions between MADS-box transcription factors and other proteins, atrophy of calyxes and petals may be caused by interference of *ZbAGL11* with the transcriptional regulation of *AtAP2* or other genes. In this study, Z*bAGL11*-OE produced multiple (>2) indefinite embryonic primordia after emasculation, and polyembryos may form during sporophytic apomixis. Based on our observations, we infer that *ZbAGL11*-OE Arabidopsis undergoes sporophytic apomixis after emasculation.

In addition to *AGL11*, class D genes also include *SHP1* and *SHP2*, which can regulate ovule development but have functional redundancy^29,30^. In Arabidopsis, *shp1* and *shp2* mutants did not show abnormal ovule development, but the *agl11* mutant showed abnormal ovule development, consistent with a need for the *AGL11* gene during ovule development^1,30,31^. An antagonistic effect has also been reported between a class A gene and a class C gene in Arabidopsis^32^. Loss of function of the C gene causes the third whorl of stamens to develop into petals and the fourth whorl of carpels to develop into petals^33^. Mutation of the class A gene causes the first whorl of flower buds and the second whorl of petals to develop into stamens and carpels, respectively^24,34^. With increased studies of flower development mechanisms, new genes have been identified with similar functions to those described in the ABCDE model, with some genes having novel functions. For example, the *CRC* and *SPT* genes have similar functions to class C genes and can promote the development of carpels and ovules^35,36^. *CAULIFLOWER (CAL)* can maintain the development of petals with mutations in class A and E genes, and in some species, the C gene does not participate in the formation of ovules^37^. Therefore, the ABCDE model does not explain all flower development types, suggesting special flower development patterns in some species^38,39^ that require modification or expansion of the model. Overall, there is a significant interplay among the members of the MADS-box transcription factor family, with both functional redundancy and antagonism^40,41^. Similar or distinct plant phenotypes can result from varied interactions of MADS-box transcription factors.

We also found that the ZbAGL11 protein interacted with ZbCYP56 and ZbACD11 through yeast two-hybrid experiments. CYP65 can catalyze the cis-trans isomerization of proline-imine peptide bonds in oligomeric oligopeptides to assist in protein folding and promote protein ubiquitin ligase activity and intracellular protein transport^42,43^. Cytochrome P450 (CYP) can regulate the synthesis of ABA^44^ and auxin^45,46^ and can also control the shape of flower organs and leaves in plants^47^. CYP protein may bind to AGL11 protein and participate in the process of ovule embryo development. Arabidopsis accelerated cell death 11 (ACD11) is a ceramide-1-phosphate transfer protein that is an intermediate regulator of phytoceramide levels in plants^48^. The protein encoded by ACD11 contains a glycolipid transfer protein (GLTP) fold, can bind and transfer ceramide-1-phosphate during membrane interaction, and can also mediate defense and programmed cell death (PCD). ACD11 and AGL11 proteins may regulate PCD during apomixis^49^. The A-E genes all encode MADS transcription factors that may combine as dimeric and tetrameric complexes and regulate plant flowering time and flower organ formation^50,51^. Overall, floral organ development is a complex regulatory process that includes many regulatory factors.

The *ZbAGL11* gene is a candidate gene for agricultural breeding, potentially with broad application potential in economic species. However, the apomictic mechanisms need to first be well characterized before the application of apomixis to agricultural breeding. In this study, the *ZbAGL11*-OE Arabidopsis seed-setting rate after detasseling was only 5%, insufficient for the large-scale production of commercial seeds. The results from this work confirm that the reproductive mode of *ZB* is sporophytic apomixis. *ZbAGL11* plays a key role in the formation of ovule heart-shaped embryos, and the overexpression of *ZbAGL11* in Arabidopsis can cause floral organ development disorders and induce seed formation for apomictic phenotypes independent of fertilization.

## Materials and methods

### Plant material

Commercial plantations of *ZB* have only female plants without male plants, and male plants of ZB are very rare in nature, making it difficult to breed offspring by sexual reproduction. *ZB* samples were harvested from the Fengxian Experimental Station of Northwest Agriculture and Forestry University (N33°59′6.55′′ E106°39′29.38′′). *ZB* (cv. ‘Hanchengdahongpao’) fruits were collected from 6-year-old Chinese prickly ash trees with uniform growth that were cultivated with regular pruning and pest control.

Plant materials were collected from the Fengxian Experimental Station, Northwest Agriculture and Forestry University, Fengxian, Shaanxi, China. Healthy, 5-year-old trees were selected with uniform growth. Unopened *ZB* flowers were selected as experimental materials and then bagged and continuously sampled at intervals of one day for collection of a total of 15 days of material. A portion of each sample was placed in FAA fixative for later cytological observation, and another portion was immediately stored in liquid nitrogen for molecular experiments. A total of 600 flowers were collected from three ZB trees at each stage.

### Total RNA extraction and cDNA synthesis

The TaKaRa MiniBEST Plant RNA Extraction Kit (TaKaRa, Dalian, China) was used for the extraction of total RNA during different fruit developmental stages. First-strand cDNA synthesis of mRNA was carried out using the PrimeScript™ RT reagent Kit (TaKaRa, Dalian, China) according to the manufacturer’s instructions, and the resulting material was used as template for the RT-qPCR reaction.

### Quantitative real-time PCR (RT-qPCR)

A CFX96 real-time PCR detection system (Bio-Rad, Hercules, CA, USA) was used to confirm the relative expression of mRNAs involved in apomixis using a reaction system of 10 μl that contained 5 μl of 2× SYBR Premix Ex Taq II (TaKaRa, Beijing, China), 1 μl of cDNA, 1 μl of each of the universal forward and reverse primers, and 2 μl of ddH_2_O. *ACTIN2* and *UBQ10* were used as reference genes for Arabidopsis^52^, and *ZBUBQ* was used as the control gene for ZB^53^. Primers were designed using Primer 5.0 (Premier, Palo Alto, CA, USA). The sequences of the primers are listed in Table S1. The RT-qPCR reaction conditions were as follows: 95 °C for 30 s followed by 40 cycles of 94 °C for 5 s, 54 °C for 30 s, and 72 °C for 45 s. Three technical replicates were performed for each sample.

### Cell ploidy assays

Young leaves, endosperms, embryos, and whole seeds of ZB were separated by a dissecting mirror, and then cell ploidy analysis was performed using a Partec CyFlow Space (Sysmex-Partec, Norderstedt, Germany) according to the instructions of the Partec CyStain^®^ UV Ploidy kit (Sysmex-Partec, Norderstedt, Germany). To confirm the embryonic origin of *ZbAGL11*-OE, we also performed flow cytometry analysis on somatic cells (leaf) of *ZbAGL11*-OE and progenies.

### Fluorescence capillary electrophoresis

To determine the genetic relationship between the parents and offspring of *ZB*, the amplification products of 12 pairs of polymorphic SSR primers were analyzed for DNA samples of three female parents and 45 offspring by fluorescence capillary electrophoresis (Table S2). In capillary electrophoresis fluorescence detection, the 5′-end of the SSR forward primers used for PCR amplification was labeled with the FAM fluorescent tag. The total PCR amplification reaction volume was 15 μl and included 1 μl of DNA (5 ng/L), 1.5 μl of 10× buffer (TaKaRa cat. no. 9151 A), 1.5 μl of MgCl_2_ (25 mmol/l), 0.3 μl of dNTPs (10 mmol/l), 0.15 μl of forward primer (10 μmol/l), 0.15 μl of reverse primer (10 μmol/l), and 0.3 μl of Taq enzyme (5 µg/µl, TaKaRa Taq cat. no. R001A). The reaction consisted of 35 cycles of 94 °C for 15 s, 55 °C for 15 s, and 72 °C for 30 s, followed by 72 °C for 3 min. PCR products were detected by capillary electrophoresis on a 3730Xl gene sequencer (ABI, Foster, CA, USA).

### Phylogenetic analysis

AGL11 protein sequences (Table S3) from 36 species were downloaded from the NCBI database and then imported into MEGA for phylogenetic tree analysis. In addition, sequences of 17 ZB MADS-box family members were collected for phylogenetic analysis.

### Plant transformation and reproductive phenotype analysis

*ZbAGL11* was inserted into a pCAMBIA2300 vector containing a gene encoding kanamycin resistance, and then the flowers of Arabidopsis ecotype Columbia (Col-0) were infected by transformed Agrobacterium. The collected seeds were cultured in a medium containing kanamycin (100 mg/l). T3 generation seeds were obtained for subsequent experiments. The *35* *S: ZbAGL11* and Col-0 *Arabidopsis thaliana* plants were cultured in an artificial climate chamber at a culture temperature of 25 ± 2 °C, an illumination intensity of 2000 lx, and a photoperiod of 16-h light/8-h dark. Flower buds were emasculated before dehiscence of the anthers according to Goetz et al.^54^. The unopened flowers were selected, and stamens and opened flowers were removed using anatomical forceps before the remaining flower cluster structure was bagged. At each stage, 100 flowers were selected from detasseled ZbAGL11-OE plants for cytological observation.

### Genetic transformation of *ZB*

The petioles were sterilized and transferred to MS medium for genetic transformation. Plant tissues were cocultured with Agrobacterium containing the *ZbAGL11* gene for 3 days in the dark (MS + TDZ 1.0 mg/l + 0.3 mg/l + 10 mmol/l AS + 100 mmol/l Bet). Subsequently, the infected material was washed three times with 500 mg/l cephalosporin, and the plant material was transferred to the differentiation medium (Cef 250 mg/l) and cultured in the dark for 2 days. The infected material was then transferred to the screening medium (Kan 25 mg/l + Cef 250 mg/l) and cultured for 30 days. After the plant material differentiated into buds, the buds were transferred to rooting medium (MS + 1.0 mg/l IBA + 0.5 NAA).

### Cytological observation of embryonic development

The collected ZB and Arabidopsis fruits were paraffin sectioned and stained with safranin O. Cytological observation of *ZB* fruit showed that the YF (7 days) was a critical period for the development of apomictic embryos. Therefore, four stages of samples were selected for apomictic research: S1 (preflowering), S2 (midflowering, 3 days after flowering), S3 (young fruit, 7 days after flowering), and S4 (fruit expansion, 15 days after flowering).

### Subcellular localization of *ZbAGL11* proteins

The full-length *ZbAGL11* gene was cloned using template cDNA from ZB fruit, and then *ZbAGL11* was inserted into the pCAMBIA2300 vector. A *ZbAGL11* green fluorescent protein (GFP) C-terminal fusion was generated and introduced into a pCAMBIA2300-GFP vector backbone under the control of the 35 S CaMV promoter. The pCAMBIA2300-GFP vector was transformed into Agrobacterium by the heat shock method, and Agrobacterium was injected into *Nicotiana benthamiana*. The fluorescence signal was observed within 12–24 h after transformation using an Olympus fluorescence microscope (Waltham, MA) and a Leica TCS SP8 confocal microscope (Buffalo Grove, IL).

### Yeast two-hybrid assays

The *ZbAGL11* sequence was inserted between the BamHI and SalI restriction sites of the yeast expression vector pGBKT7, resulting in the pGBKT7-*ZbAGL11* vector. To verify the self-activation of the bait vector, the pGBKT7-*ZbAGL11* vector was transferred into yeast Y187, cultured in SD/-Trp solid medium; single colonies were picked, and the colonies were verified on X-α-gal SD/-Trp/-Ade and SD/-Trp/-His media. According to the manufacturer’s instructions, the Make Your Own Mate & Plate^™^ Library System Kit (Takara, Beijing) was used to construct a library of mixed cDNAs of *ZB* roots, stems, leaves, flowers, and fruits for use in the yeast two-hybrid assay. The yeast two-hybrid library plasmid and pGBKT7-*ZbAGL11* were cotransformed into yeast Y187, and the transformed yeast was cultured on SD/-Trp/-Leu medium at 30 °C for 2–3 days. After growth, white colonies were selected and transferred to SD/-Ade/-His/-Leu/-Trp quadruple deficiency medium containing X-α-gal for screening. Colonies that turned blue on the medium indicated an interaction between the genes expressed on the two constructs. Separately, pGBKT7-*p53* and pGADT7-T were cotransformed as positive controls, and pGBKT7-*Lam* and pGADT7-T were cotransformed as negative controls. To verify the reliability of the interaction between genes detected by yeast two-hybrid screening, the genes were then cloned into the opposite vector, and the interaction was confirmed.

## Supplementary information

Supplemental material
