# Supplementary material for: ZbAGL11, a class D MADS-box transcription factor of Zanthoxylum bungeanum, is involved in sporophytic apomixis
Source: Hortic Res. 2021 Feb 1;8:23. doi: 10.1038/s41438-020-00459-x (PMC7848008; doi:10.1038/s41438-020-00459-x)
Supplement: Supplementary file 1 — Supplemental material [file 41438_2020_459_MOESM1_ESM.docx]

**Supplemental material**

**
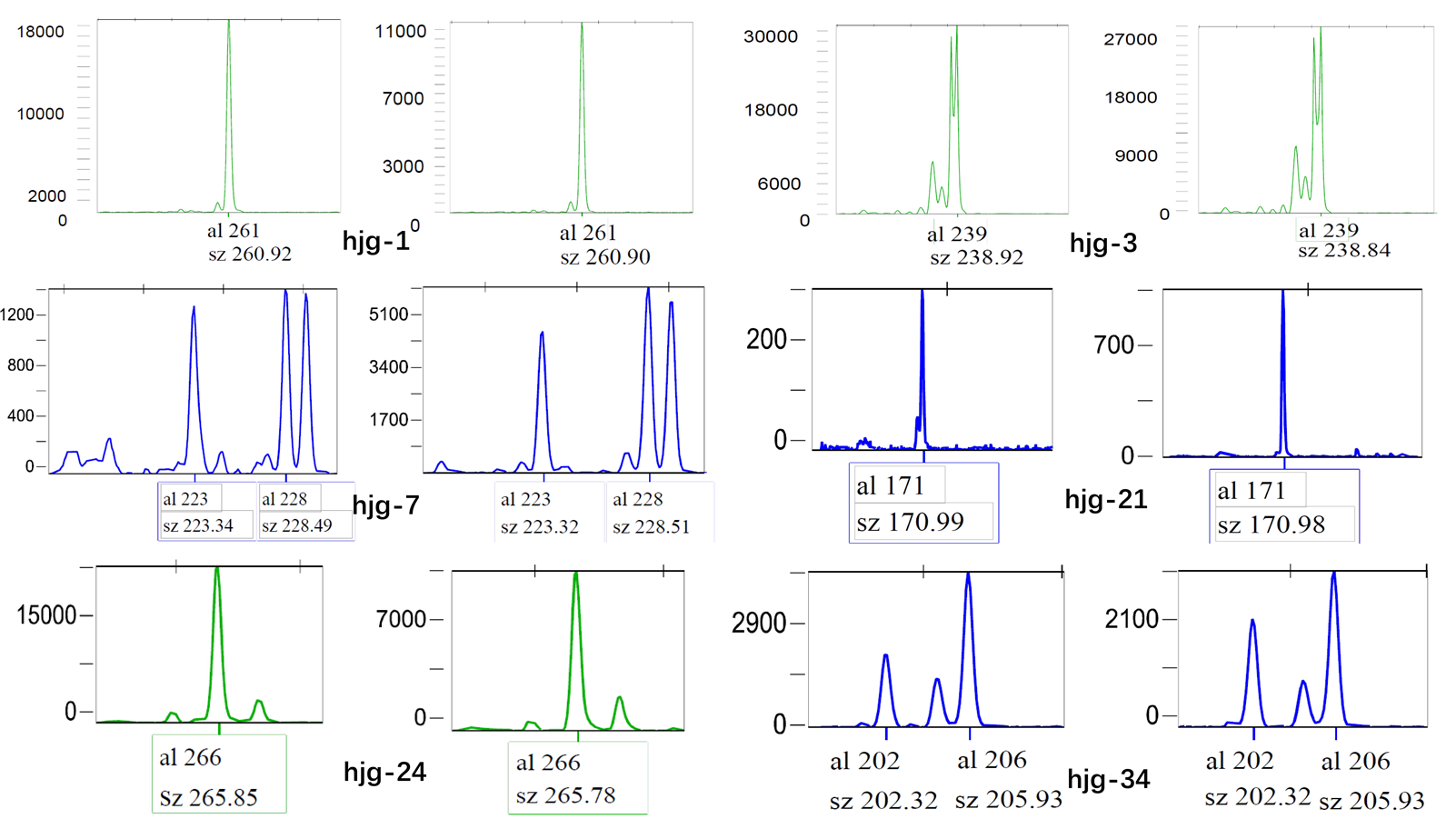
**

**Fig. S1-1** **Detection of partial PCR products by capillary electrophoresis.**

**
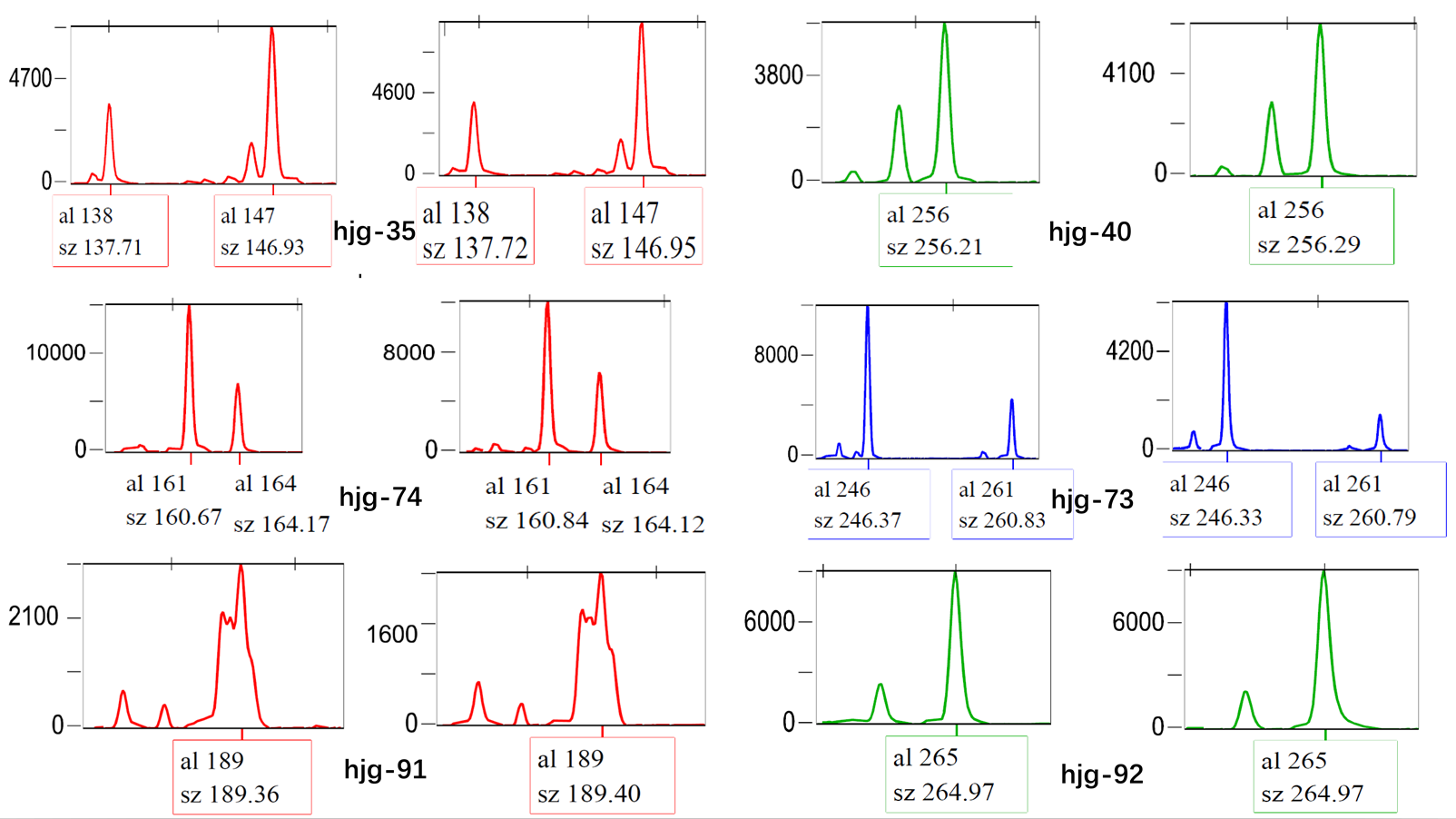
**

**Fig. S1-2 Detection of partial PCR products by capillary electrophoresis.**

**
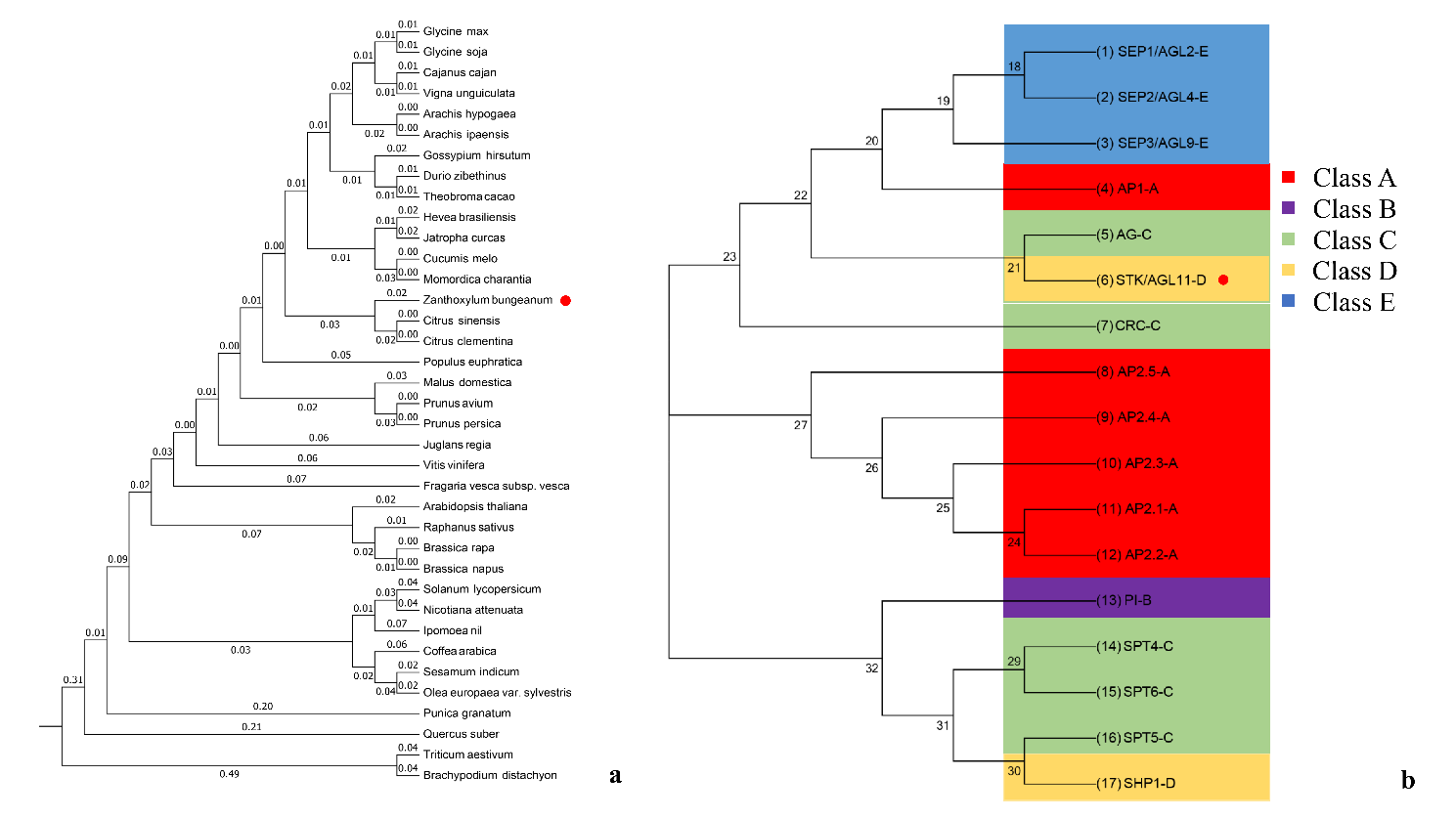
**

**Fig. S2** **Phylogenetic tree analysis of MADS-box transcription factors.** **(a)** Phylogenetic tree of *AGL11*. (**b)** Phylogenetic tree of MADS-box transcription factors in *Zanthoxylum bungeanum.*

**
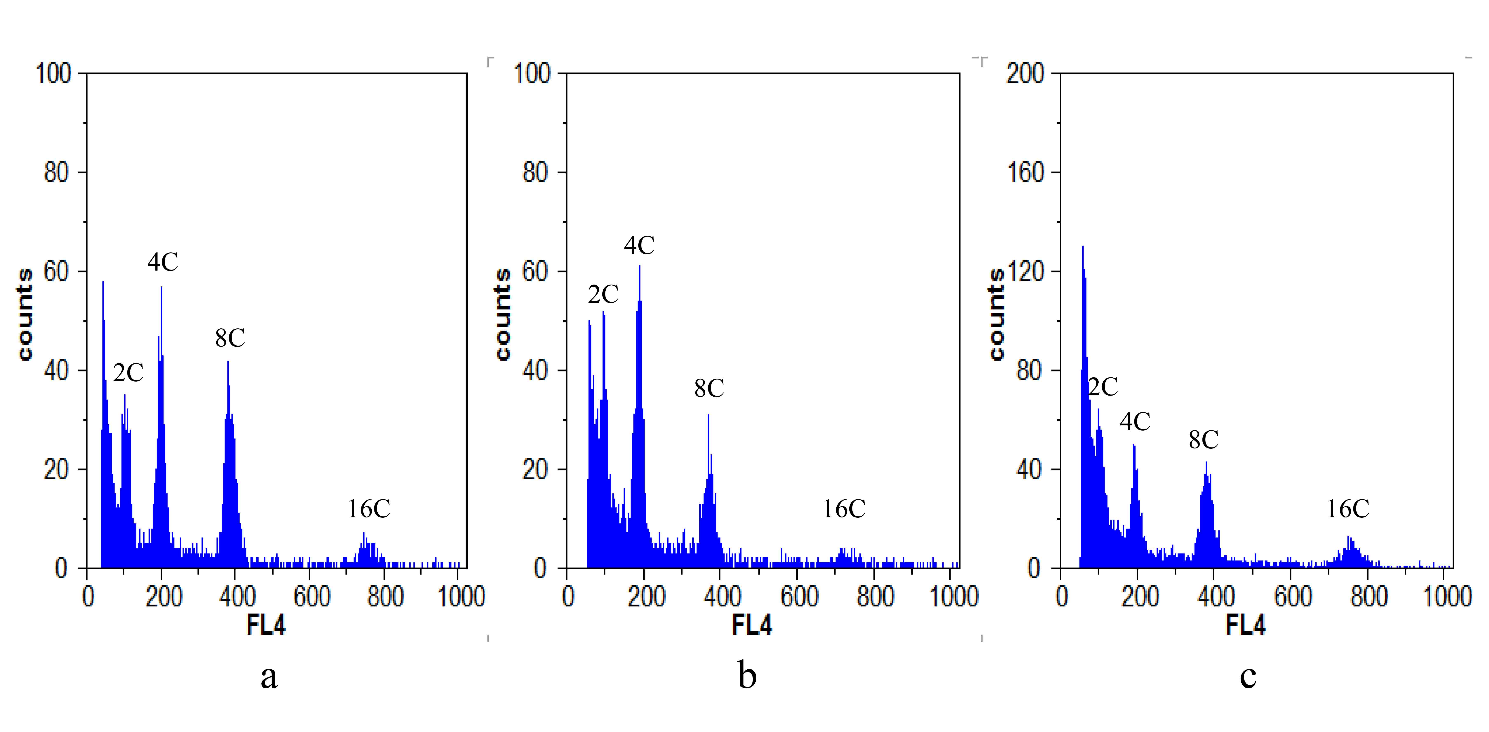
**

**Fig. S3** **Ploidy Analysis of *ZbAGL11*-OE and progeny of *ZbAGL11*-OE.** **(a)** Wild type Arabidopsis, **(b)** *ZbAGL11*-OE, (**c)** progeny of *ZbAGL11*-OE.

**Table S1 Primer sequences for RT-qPCR.**

| Gene categories | Annotation | ID | 5’-3’ Primer Sequences (F) | 5’-3’ Primer Sequences (R) | Amplicon Length (bp) | Primer Efficiency |
| --- | --- | --- | --- | --- | --- | --- |
| Class A | *AP1* | NM_105581.3 | CTCTCTCATCAGCCATCTCCTT | TTCGAGATCATTCCTCCTCATT | 87 | 102.6% |
| Class A | *AP2* | NM_001204009.1 | AGCTCGAATGGGTCAATTCTTA | TCTTTGCCGTTACATTTGATTG | 114 | 95.3% |
| Class B | *AP3* | NM_115294.6 | GAGGCTAGGTGAGTGTTTGGAC | TCGATCTGATTCCCAAGAGATT | 123 | 98.7% |
| Class B | *PI* | NM_122031.4 | GGATCTTGGTGCTATGTTGG | CTCGACAGCCATCAGATTTT | 190 | 90.6% |
| Class C | *AG* | NM_001341309.1 | ACAAGAATCAGCCAAATTGC | TTTGGGAGACATTGACCCTA | 94 | 103.2% |
| Class C | CRC | NM_105585.3 | TTAGCCTCACCCTTCAGATG | CTTCTCAGGAGGTTTGACGA | 137 | 97.2% |
| Class D | *AGL11* | NM_001340645.1 | ATTCTCAGGATGGGAAGAGG | GAAAACAATGAGCGCAACTT | 153 | 98.1% |
| Class D | *SHP1* | NM_115740.3 | TAACATGTACCTGCGAGCAA | CCGATTATAATGCTGCGACT | 136 | 101.7% |
| Class D | *SHP2* | NM_129844.5 | GCTGAGGTTGCTCTTGTCAT | TCTGAATGTCCCGAATCTGT | 196 | 92.3% |
| Class E_1_ | *AGL2* (*SEP1*) | NM_001125758.2 | GGAAGGTGGTGAACAGAATG | GCTCTGAGCATACCGGATTA | 155 | 96.0% |
| Class E_2_ | *AGL4* (*SEP2*) | NM_111098.4 | ATTGCCTATGGACATCCTCA | ATGTAGCCGTTTCCTTGTTG | 152 | 99.2% |
| Class E_3_ | *AGL9* (*SEP3*) | NM_180622.3 | AGTAAGGAACGCATGCTGAC | GACGACCGTAGTGATCAACC | 118 | 98.1% |
| Class E_4_ | *AGL3* (*SEP4*) | NM_179599.2 | TTTTCTCTAACCGTGGCAAG | GCCTTTGTGAATGTTGAAGG | 192 | 91.8% |
| Reference gene 1 | *ACTIN2* | NM_112764.4 | CTTGCACCAAGCAGCATGAA | CCGATCCAGACACTGTACTTCCTT | 68 | 94.4% |
| Reference gene 2 | *UBQ10* | NM_178968.5 | GGCCTTGTATAATCCCTGATGAATAAG | AAAGAGATAACAGGAACGGAAACATAGT | 61 | 97.5% |

**Table S2 SSR capillary electrophoresis primers.**

| Primer Name | Primer Seqence (5'-3') | Primer Seqence (5'-3') | Repeat Motif | Production Size | Na | PIC | Ho | He |
| --- | --- | --- | --- | --- | --- | --- | --- | --- |
| hjg-1 | F: TGTCTTCGCCTTCCATTCTC | R:CGAGCACCAACCCTAACAAT | (AG)10 | 272 | 3 | 0.48 | 0.13 | 0.57 |
| hjg-3 | F: GCTTCGTCAGGCAGAAACTC | R: CAAAATCGGTCTTCGCTTTC | (AG)8 | 238 | 4 | 0.59 | 0.64 | 0.68 |
| hjg-7 | F: TCACTCCTATGCCTCCTTGG | R:TGATCTTGGTGCCACAGGTA | (TA)10 | 219 | 5 | 0.58 | 0.40 | 0.64 |
| hjg-21 | F: GGCCGCCTGAAGAATACAT | R:TTCGGCTAACCAAACAAACC | (ATT)8 | 142 | 3 | 0.30 | 0.38 | 0.34 |
| hjg-24 | F: AACGCGCCATTTCATATTTC | R:AGAGCATTGAGCCTCGTTGT | (AT)8 | 189 | 5 | 0.73 | 0.54 | 0.80 |
| hjg-31 | F: GTACAAGCGATGCGACAGAA | R:AGTGCGTGACTCGAACAGTG | (TA)14 | 256 | 2 | 0.36 | 0.00 | 0.49 |
| hjg-34 | F: CCAACATCAAAGAAACGCAA | R: CATAATTCCTAGGTTGGCCG | (AAT)6 | 163 | 2 | 0.37 | 0.00 | 0.51 |
| hjg-35 | F: GCTGTGAACATGAAATCGGA | R:TCGCGTGAAATAGAATGTCG | (TA)13 | 189 | 3 | 0.40 | 0.33 | 0.46 |
| hjg-40 | F: GTCGTCAAATGAACCGTGTG | R:AATCGATTCGGTGTGTGGAT | (TATATG)5 | 204 | 4 | 0.50 | 0.40 | 0.61 |
| hjg-73 | F: GGATGCCAATCCTTCACACT | R:TGAATAGTACTTGGGGGCCA | (ATT)9 | 263 | 3 | 0.59 | 0.23 | 0.69 |
| hjg-74 | F: TCCACGTCAACTCCAAACAA | R:GACTCAACTGTCGGTGCTCA | (AT)9 | 259 | 3 | 0.49 | 0.07 | 0.60 |
| hjg-91 | F: CCATGCAACAGCGATTCTAA | R:TCCACACACATGTCAAACACA | (TG)9 | 262 | 5 | 0.47 | 0.43 | 0.52 |
| hjg-92 | F: CGCTGCCATTATTTGCTGTA | R: TGGTGGCACTTAGCAGTGAG | (ATA)12 | 249 | 7 | 0.75 | 0.73 | 0.81 |

Note: Na:Number of alleles; PIC:Polymorphic information content; Ho:Heterozygosity observed; He: Hexp (expected heterozygosity).

**Table S3 Protein sequence information of AGL11 from 37 species.**

| Species | GeneBank ID | Length(bp) |
| --- | --- | --- |
| *Zanthoxylum bungeanum* | MT276586 | 222 |
| *Solanum lycopersicum* | NP_001234194.1 | 198 |
| *Glycine max* | NP_001236130.1 | 222 |
| *Triticum aestivum* | ABF57916.1 | 228 |
| *Vitis vinifera* | NP_001268118.1 | 223 |
| *Arabidopsis thaliana* | NP_001319889.1 | 329 |
| *Brachypodium distachyon* | NP_001288317.1 | 229 |
| *Hevea brasiliensis* | XP_021686558.1 | 223 |
| *Punica granatum* | XP_031394190.1 | 179 |
| *Arachis hypogaea* | XP_025657298.1 | 220 |
| *Gossypium hirsutum* | NP_001314587.1 | 224 |
| *Cajanus cajan* | XP_020240315.1 | 222 |
| *Citrus sinensis* | XP_024953931.1 | 224 |
| *Coffea arabica* | XP_027093918.1 | 228 |
| *Citrus clementina* | XP_024044078.1 | 224 |
| *Durio zibethinus* | XP_022718375.1 | 222 |
| *Juglans regia* | XP_018841133.1 | 222 |
| *Arachis ipaensis* | XP_020959102.1 | 220 |
| *Populus euphratica* | XP_011031534.1 | 224 |
| *Fragaria vesca subsp. vesca* | XP_011461174.1 | 248 |
| *Sesamum indicum* | XP_011096010.1 | 222 |
| *Glycine soja* | XP_028230285.1 | 222 |
| *Vigna unguiculata* | XP_027921542.1 | 222 |
| *Olea europaea var. sylvestris* | XP_022852796.1 | 159 |
| *Prunus avium* | XP_021831176.1 | 221 |
| *Brassica rapa* | XP_009113584.1 | 220 |
| *Cucumis melo* | XP_008459658.1 | 226 |
| *Prunus persica* | XP_020416253.1 | 221 |
| *Nicotiana attenuata* | XP_019249035.1 | 228 |
| *Raphanus sativus* | XP_018452528.1 | 230 |
| *Quercus suber* | XP_023928415.1 | 211 |
| *Ipomoea nil* | XP_019176799.1 | 231 |
| *Jatropha curcas* | XP_012073508.1 | 222 |
| *Brassica napus* | XP_013656088.1 | 230 |
| *Momordica charantia* | XP_022149992.1 | 223 |
| *Theobroma cacao* | XP_017980812.1 | 222 |
| *Malus domestica* | XP_028950486.1 | 222 |
